# *Elymus nutans* genes for seed shattering and candidate gene-derived EST-SSR markers for germplasm evaluation

**DOI:** 10.1186/s12870-019-1691-4

**Published:** 2019-03-13

**Authors:** Yongqiang Zhao, Junchao Zhang, Zongyu Zhang, Wengang Xie

**Affiliations:** State Key Laboratory of Grassland Agro-Ecosystems, Key Laboratory of Grassland Livestock Industry Innovation, Ministry of Agriculture and Rural Affairs, College of Pastoral Agriculture Science and Technology, Lanzhou University, Lanzhou, 730020 People’s Republic of China

**Keywords:** *Elymus*, Seed shattering, Comparative transcriptome, Candidate genes, Gene-derived EST-SSR marker

## Abstract

**Background:**

*Elymus nutans* and *E. sibiricus* are two important forage grasses of the genus *Elymus*. But they are difficult to grow for commercial seed production due to serious seed shattering. We conducted a comparative transcriptome analysis of abscission zone to find possible transcription changes associated with seed shattering, explore candidate genes involved in seed shattering and identify candidate gene-based EST-SSR markers for germplasm evaluation.

**Results:**

cDNA libraries from abscission zone (AZ) and non-abscission zone (NAZ) tissues of *E. nutans* were constructed and sequenced. A total of 111,667 unigenes were annotated and 7644 differentially expressed transcripts (DETs) were predicted, corresponding to 6936 up-regulated in AZ and 708 down-regulated in NAZ. We identified 489 candidate genes related to transcription factor, cell wall hydrolysis or modification, hydrolase activity, phytohormone signaling and response, lignin biosynthesis, and signal transduction or protein turnover. Eleven similar candidate genes involved in polygalacturonase activity, hydrolase activity, and mitogen-activated protein kinase were up-regulated in the abscission zone of the two *Elymus* species*,* suggesting these genes may have specific function for abscission zone development and seed shattering. A total of 67 polymorphic EST-SSR markers were developed and characterized based on the sequences of these candidate genes. Fourteen polymorphic EST-SSR primers were finally used to study genetic diversity in 48 *E. nutans* genotypes with contrasting seed shattering habit. The dendrogram based on molecular data showed that most accessions with similar seed shattering degree tended to group together.

**Conclusions:**

The expression data generated from this study provides an important resource for future molecular biological research. Many DETs were associated with abscission zone development, and EST-SSR loci related to candidate genes may have potential application in identifying trait-associated markers in *E. nutans* in the future.

**Electronic supplementary material:**

The online version of this article (10.1186/s12870-019-1691-4) contains supplementary material, which is available to authorized users.

## Background

Seed Shattering is an important adaptive trait for seed dispersal in wild plants, but is also a major cause of yield loss in many cereal crops and forage grasses during harvest [1]. During early domestication of major crops like rice, wheat and barley, low seed shattering has been selected as one of the most important agronomic traits [2]. In comparison, breeding objectives of forage grasses mainly focus on biomass yield, forage quality and stress tolerance. Seed shattering improvement of many forage grasses has therefore seriously lagged behind crops plant, despite seed shattering is a commonly observed trait in many forage varieties and wild grass species [2–4]. Previous research showed that both seed and herbage yield could be increased through the selection for high fresh weight at seed harvest [5]. In addition, increased seed retention did not influence forage quality and suggested the selection for seed retention would be one of the important breeding objectives for forage grasses with high seed shattering degree [6].

*Elymus nutans* and *E. sibiricus,* which belong to the genus *Elymus* (Poaceae: Triticeae), are two important perennial herbaceous plants mainly distributed in the high altitude regions of Western and Northern China [7]. As the two *Elymus* species have good forage yield and quality as well as excellent cold and drought tolerance, they have been widely used as forage crops in cultivated pastures and natural grassland. Despite their economic importance, the two *Elymus* species are difficult to grow for commercial seed production due to serious seed shattering. In previous studies, we found high seed shattering degree in wild accessions and cultivars of the two *Elymus* species [1, 8]*.* Indeed, seed shattering can cause up to 80% seed yield losses if harvesting is delayed due to some adverse conditions [3]. Therefore, selection for seed retention and improvement of seed shattering for the two species could be important objectives in breeding programme.

Seed shattering is a complex process governed by highly coordinated changes in plant cell structure, metabolism and putative gene expression. Previous studies showed seed shattering is highly associated with the formation, development and degradation of abscission layers that located in the flower and pedicel junction [9, 10]. In many cereals, seed retention results from loss of the abscission layers [9]. Many forage grasses possess clear, defined abscission layers at the heading stage [6, 11]. Seed shattering variation among wild plants and cultivars results from different degradation degree of abscission layers. A previous study in *E. sibiricus* showed a higher degradation degree of abscission layers in high seed shattering genotype [10]. Meanwhile, seed shattering habit is a complex trait which is controlled and regulated by many genes [12]. To date, many shattering genes have been reported in several crops. Major genes for seed shattering were identified and cloned in rice, including *SH4* [13], *qSH1* [14], *OsCPL1* [15], *SHAT1* [16], and *SH5* [17]. *SH4*, a major seed shattering QTL, encodes a transcription factor with Myb3 DNA binding domain responsible for a reduction in seed shattering [13]. *qSH1* is another important seed shattering QTL, which encodes a BEL1-type homeobox gene and regulates abscission zone formation [14]. In Arabidopsis, floral organ abscission could be regulated by the overexpression of wheat BEL1-like gene *TaqSH1* [18]. A major wheat domestication gene *Q* on chromosome 5A regulates plant architecture and seed dispersal [19]. *Shattering 1* (*Sh1*), which encodes YABBY transcription factor, regulates seed shattering in Sorghum [20]. In maize, *Sh1* orthologous genes on chromosome 1 and 5 have been identified as major QTLs related to seed shattering [19].

In comparison, studies of seed shattering in forage grasses are limited. A major-effect seed retention QTL on LG6a was identified in hybrid *Leymus* (Triticeae) wildryes [21]. A MIKC-type MADS-box gene *EnWM8* was cloned in *E. nutans* [18]. Our previous transcriptome study of abscission zone in *E. sibiricus* identified more than 7000 differentially expressed genes, and indicated many putative genes involved in hrdrolytic enzyme activity, phytohormone signaling, and lignin biosynthesis were up regulated in abscission zone tissue of high seed shattering genotype [10]. But it is unclear whether these *E. sibiricus* candidate genes could be found in other *Elymus* species like *E. nutans,* suggesting that identifying the genes which regulate seed shattering among different *Elymus* species is critical.

To better understand the mechanism for seed shattering in *Elymus* species, explore the putative candidate genes related to seed shattering, and identify candidate gene-based EST-SSR markers for germplasm evaluation, we carried out a comparative transcriptome analysis of abscission zone in the two *Elymus* species. The results of this study will lead to a better understanding of seed shattering, and would be helpful for genetic improvement and marker assisted selection of seed shattering for the two *Elymus* species.

## Methods

### RNA extraction

The histological analysis, including logitudinal and cross section, and scanning electron microscopy was used to examine the pedicel junctions before RNA extraction. Histological analysis of pedicel structure was carried out at 14 days after heading (DAH) for *E. nutans*. To detect the abscission layer development at different stages, the pedicel tissues at 21 and 28 DAH were selected for *E. sibiricus*. Scanning electron microscopy was used to examine the pedicel junctions after detachment of seeds. Histological analysis was carried out according to the methods described by Zhao et al. [11]. Abscission zone (AZ) and non-abscission zone (NAZ) tissues of *E. nutans* were collected at 14 days after heading (DAH). Abscission pathway includes four major steps: abscission zone formation and development, response to abscission signals, activation of abscission, and differentiation of the abscission layer [10]. Seed shattering was commonly visible at 21 DAH, many genes involved in seed shattering should be activated before this time point, therefore, tissues at 14 DAH were collected. Abscission zone (AZ) consisted of an approximately 1- mm region of the pedicel and 1.5 mm of the flower [13, 15]. The rest region of each pedicel is referred to as non-abscission zone (NAZ). Approximately 30 mg of tissue was collected for each replicate. Total RNA was extracted from each tissue according to the manufacturer’s instructions of Plant total RNA Kit (TIANGEN, Beijing, China). RNA concentration and quality were measured using an Agilent 2100 Bioanalyzer (Agilent Technologies, Inc. Waldbronn, Germany). The test was carried out with three biological replicates.

### Construction of cDNA library and RNA-Seq

High quality RNA samples from AZ and NAZ tissues were sent to Breeding Biotechnologies Corporation (Yangling, China) for cDNA library construction and transcriptome sequencing. The poly (A) mRNA was enriched with magnetic Oligo (dT)-rich magnetic beads and then broken into short fragments. Taking these cleaved mRNA fragments as templates, the first cDNA strand was synthesized by using random hexamer-primer. DNA polymerase I (New England BioLabs) and RNase H (Invitrogen), buffer and dNTPs were added to synthesize the second strand. The resulting cDNAs were then subjected to end-repair and phosphorylation using T4 DNA polymerase and Klenow DNA polymerase. Then, an‘A’base was inserted as overhang at the 3’ ends of the repaired cDNA fragments. After that, the cDNA fragments were ligated to sequencing adaptors, and the DNA fragments with required length were purified by agarose gel electrophoresis and gathered by PCR amplification. Finally, purified cDNA library was subjected to sequence by the Illumina HiSeq™ 4000 (Illumina Inc. USA) using the Chrysalis 36 cycles v 3.0 sequencing kit, with one lane of 2 × 101 bp reads from both ends of the fragments (“paired ends”) with 180 bp insert distance for assembly.

### Sequence filtering, De novo assembly, and annotation

The high quality clean reads were obtained from raw data by filtering adaptor sequences, duplicated sequences, and low-quality reads with ambiguous ‘N’ bases and with Q-value ≤20.. Then, De novo transcriptome assembly of the quality reads were performed to obtain unigenes using trinity program [22]. To annotate the assembled unigenes, the unigene sequences were queried using BLASTX (E-value ≤1e-5) against various databases like the NCBI non-redundant protein sequence (Nr), Gene Ontology (GO), Cluster of Orthologous Groups (COG), euKaryotic Orthologous Groups (KOG), Protein family (Pfam), Annotated protein sequence database (Swiss-Prot), and Kyoto Encyclopedia of Genes and Genomes (KEGG). GO annotation regarding the biological process, cellular component and molecular function were obtained using the Blast2GO software [23], and the GO functional classification of unigenes was performed using the WEGO software [24].

### Differentially expressed transcripts (DETs) analysis

Transcripts were mapped to the assembly, and the counts for each transcript was calculated using SOAPaligner, the unigene expression level was determined using the Fragments Per Kilobase per Million fragments mapped (FPKM) method described by Mortazavi et al. [25]. The formula log_2_ (FC) was used to calculate the the transcript fold-change, and the correction for multiple tests used the false discovery rate (FDR) control method [26]. FDR ≤ 0.01 and the absolute value of log2 (FC) ≥ 1 were set as the threshold to identify significant DETs. The DETs were clustered using STEM software with a *p* ≤ 0.05 [27]. The GO enrichment analysis was conducted using agriGO [28]. The KEGG pathway enrichment analysis of the DETs was performed using KOBAS 2.0 [29].

### Validation of RNA-seq data by quantitative real-time PCR (qRT-PCR)

To quantitatively determine the reliability of transcriptome data, the expressions of sixteen randomly selected DETs were analyzed using the qRT-PCR method. A portion of the pooled total RNA used for the RNA-Seq analysis was used to make cDNA for the qRT-PCR. The qRT-PCR was performed according to the SYBR Premix Ex Taq™ II quantitative PCR system (Takara, Dalian), following the manufacturer’s instructions, and reactions occurred on a Bio-Rad iQ5 real-time PCR instrument (Bio-Rad, Hercules, CA, USA). Gene-specific primers were designed using Primer Express software (Applied Biosystems) and are shown in Additional file 1: Table S1. Expression levels of these DETs were calculated relative to reference gene *GAPDH* using the 2^-ΔΔCt^ method [30]. Each qRT-PCR analysis was performed in triplicate, and the experiments were performed on three biological replicates.

### Gene-based EST-SSR marker development

SSRs were detected in differentially expressed gene sequences using the Simple Sequence Repeat Identification Tool Program. The EST-SSR primers were designed using Primer3 (http://bioinfo.ut.ee/primer3-0.4.0/), and the designed EST-SSR primers were synthesized by Shanghai Sangon Biological Engineering Technology (Shanghai, China).

### Plant material for genetic diversity analysis

A total of 12 *E. nutans* accessions (4 individuals of each accession) including two contrasting panel (high seed shattering and low seed shattering) were used for candidate genes-based EST-SSR marker validation and genetic diversity analysis (Table 1). Totally, 48 individual plants were grown in the field plots in the experimental station at Lanzhou University, Yuzhong, Gansu, China (latitude 35°34′ N, longitude 103°34′ E, elevation 1720 m). The level of seed shattering of each accession was determined by measuring the breaking tensile strength (BTS) required to detach the seeds from the pedicels [7]. It has a spike containing 15–28 spikelets. Twenty randomly chosen spikelets of each plant were measured at seed maturity stage, and their average BTS values were calculated.

**Table 1:**
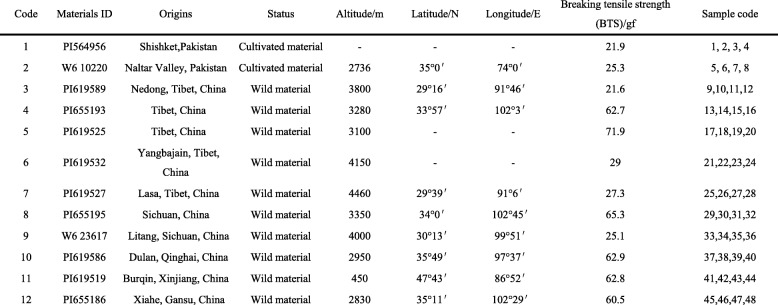
*Elymus nutans* accessions used for genetic diversity analysis, their origin, status, geographical information and seed shattering rate

### DNA extraction and polymerase chain reaction (PCR) amplification

Fresh leaf tissues of each individual were collected, lyophilized, and used for DNA extraction using the SDS (sodium dodecyl sulfate) method [31]. DNA quantity and quality were determined using the NanoDrop ND1000 spectrophotometer (Thermo Scientific, USA) and agarose gel electrophoresis. Then DNA samples were diluted to 25 ng/μL and stored at − 20 C. The PCR amplification, SSR genotyping, and the electrophoresis process were performed according to the methods described by Zhang et al. [7].

### Data analysis

The clear and reproducible bands amplified from 14 primers were scored as present (1) and absent (0), the binary matrix data was used for further genetic diversity analysis. Polymorphism information content (PIC) value of each primer was calculated according to a previously reported method: PIC = 1 – *p*^2^ – *q*^2^, where *p* is the frequency of present band and *q* is the frequency absent band [7]. POPGENE 1.31 program was used to analyze the pairwise genetic differentiation and genetic distance among these individuals [32]. Based on Jaccard’s genetic similarity coefficient, a dendrogram was constructed using the unweighted pair group method with arithmetic mean (UPGMA) [33]. The genetic structure of the 48 *E. nutans* individuals was analyzed using STRUCTURE v 2.3.4 software, and the method was described by Zhang et al. [7].

## Results

### Transcriptome sequencing and assembly

**Fig. 1:**
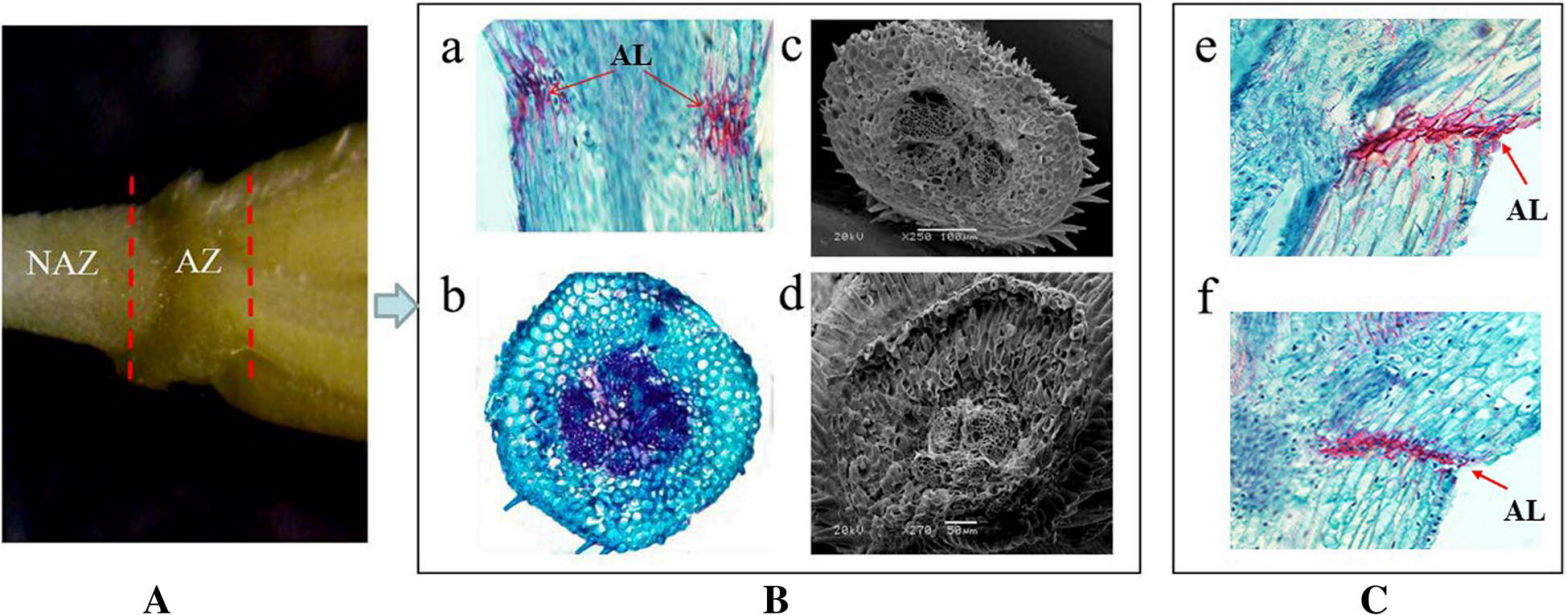
Characterization of seed shattering and seed abscission zone histology. **a**, abscission zone (AZ) consisted of an approximately 1- mm region of the pedicel and 1.5 mm of the flower. The rest regions of pedicel is referred to as non-abscission zone (NAZ). **b**, longitudinal section (**a**) and cross section (**b**) of AZ. Abscission layers (AL) could be stained dark red by safranin. Scanning electron photos of pedicel- seed junction after detachment of seed, c, pedicel region, d, seed region. C, longitudinal section of abscission layers at 21 DAH (e) and 28 DAH (f) in *E. sibiricus*

The abscission layer (AL) was found in *E. nutans* and *E. sibiricus* by histological analysis of abscission zone. Anatomical investigation with longitudinal sections showed the AL was located in the rachilla just below each floret, and occurred on both sides of the vascular bundle in the two *Elymus* species. Degradation of the abscission layer was observed in *E. sibiricus* at 21 DAH (Fig. 1). To identify candidate genes differentially expressed in the non-abscission zone (NAZ) and abscission zone (AZ) of *E. nutans*, six cDNA libraries were constructed from NAZ and AZ tissues RNA samples with three biological replicates, and then sequenced using the Illumina HiSeq™ 4000 platform. After cleaning and checking, we obtained a total of 36.64 Gb clean data, corresponding to 60.93 and 60.21 million clean reads in NAZ and AZ, respectively (Table 2). A total of 137,888 unigenes were identified with the N50 length of 1191 bp, of which 111,667 unigenes were annotated in at least one database after Blast searches of the GenBank COG, GO, KEGG, KOG, Pfam, Swiss-Prot, and Nr databases (Table 3, 4). A total of 21.05 Gb clean data were obtained from *E. sibiricus* abscission zone transcriptome data (https://www.ncbi.nlm.nih.gov/biosample/6545378), corresponding to 42.38 and 42.34 million clean reads in AZ-21 (21 days after heading) and AZ-28 (28 days after heading), respectively.

**Table 2:**
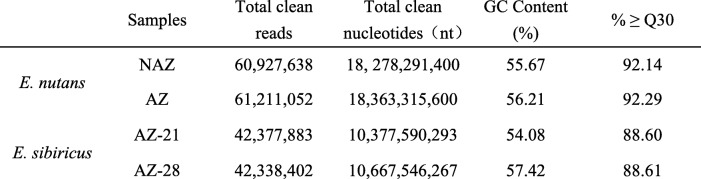
Summary of the sequence data analysis

**Table 3:**
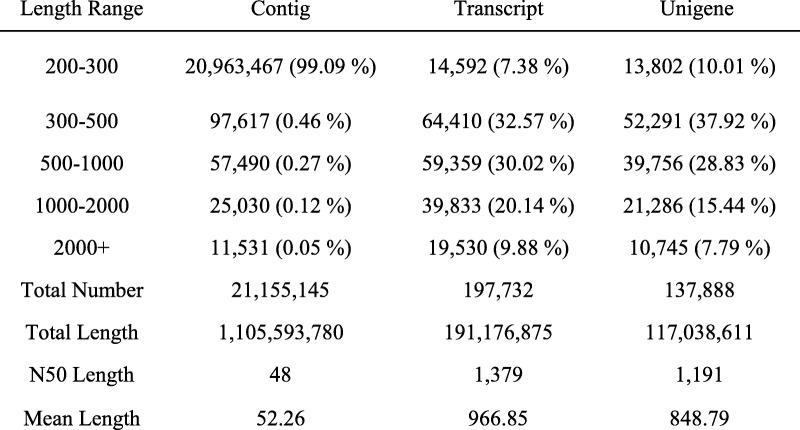
Statistics of Unigene library of *E. nutans*

**Table 4:**
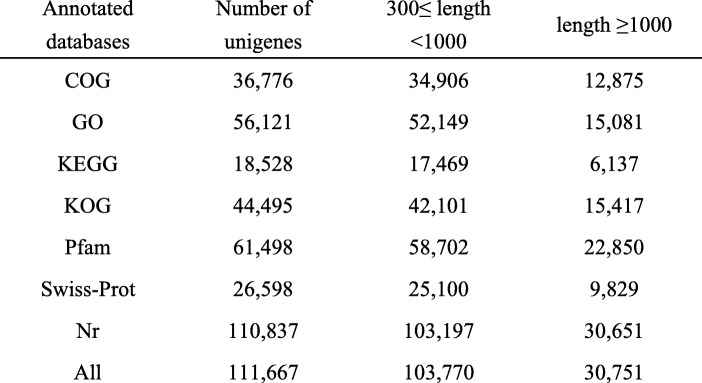
BLAST analysis of the non-redundant unigenes against public databases for *E. nutans*

### RNA-seq expression validation by qRT-PCR

**Fig. 2:**
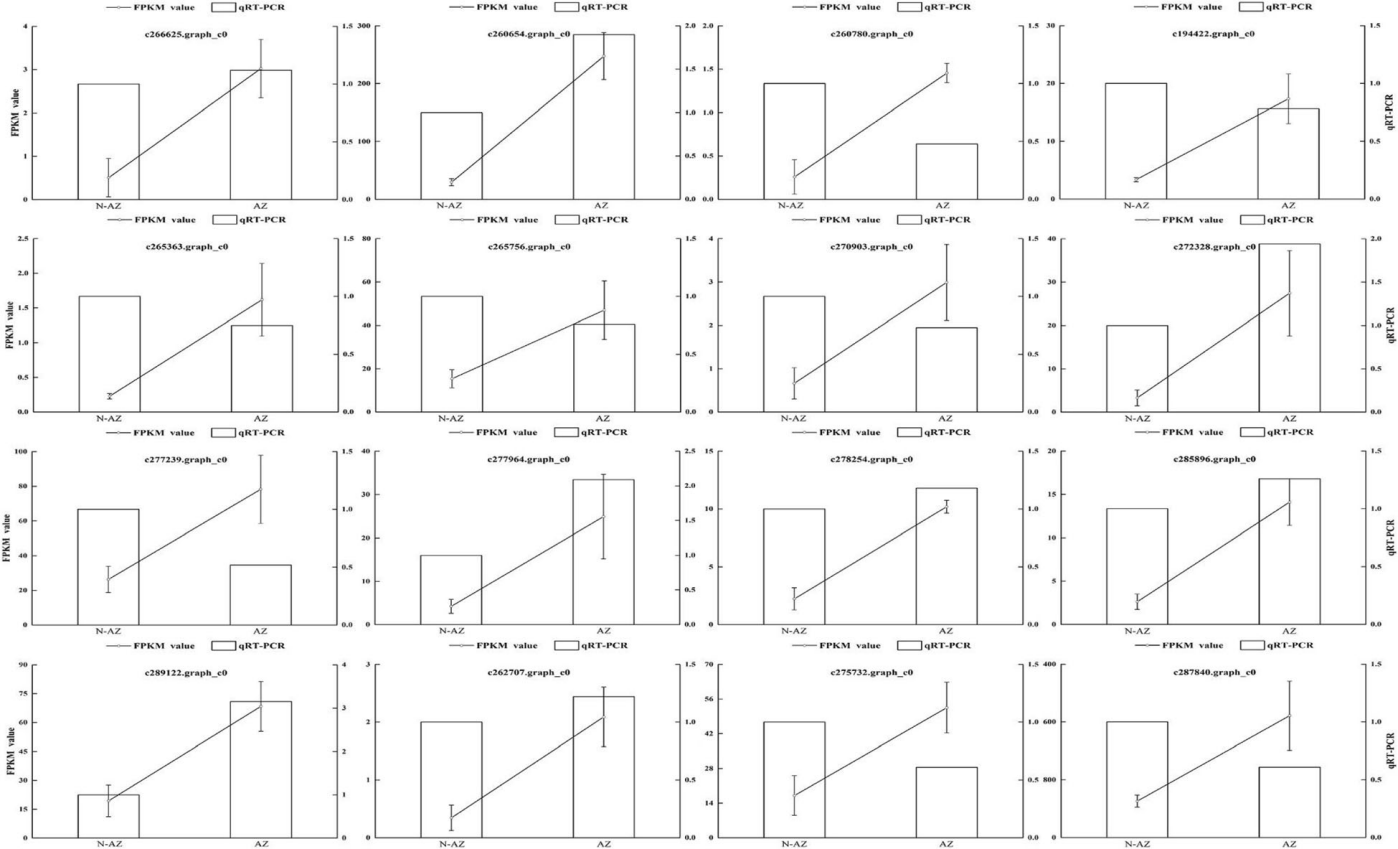
qRT-PCR validations of RNA-seq data. Expression profiles of 16 selected genes as determined by RNA-seq and qRT-PCR. Data was collected from abscission zone (AZ) and non abscission zone (NAZ). The left-hand y-axis indicates FPKM value. The right-hand y-axis indicates relative expression level. Bars indicate the mean values ± standard deviation

To confirm the reliability of our transcriptome data, the expression fold change of 16 randomly selected transcripts were determined using quantitative real-time PCR (qRT-PCR) and further compared with RNA-Seq data. In this study, we used non-abscission zone (NAZ) as a benchmark for relative expression analysis. Based on our results, eight genes were up-regulated in abscission zone (Fig. 2). A positive correlation coefficient (*r* = 0.72, *p* < 0.05) was obtained by a linear regression analysis, suggesting that the expression profiles of these 16 transcripts determined by qRT-PCR were generally consistent with the RNA-seq data.

### Differentially expressed transcripts (DETs) and annotation

To investigate the changes in gene expression and explore the putative genes involved in seed shattering in two *Elymus* species. The differentially expressed transcripts were identified between *E. nutans* non-abscission zone (NAZ) and abscission zone (AZ), and different development stages of abscission zone (21 and 28 days after heading) in *E. sibiricus*, respectively. In *E. nutans*, we identified 7644 differentially expressed transcripts (DETs), corresponding to 708 down-regulated in NAZ and 6936 up-regulated in AZ. In *E. sibiricus*, we identified 2681 differentially expressed transcripts (DETs) in abscission zone, of which 1458 unigenes were up-regulated in AZ at 21 days after heading, and 1223 were down-regulated in AZ at 28 days after heading. Then these DETs were searched against seven public databases, 7303 and 2214 DETs were annotated in seven public databases for *E. nutans* and *E. sibiricus*, respectively (Table 5).

**Table 5:**
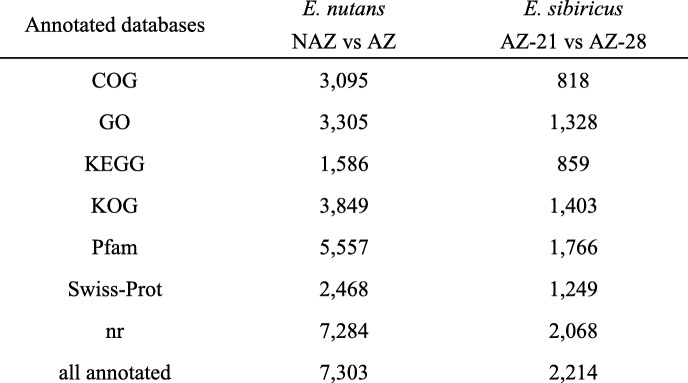
Statistical table of differently expressed transcripts (DETs), with annotation

To reveal the significantly enriched GO terms in the DETs, a GO enrichment analysis of the functional significance was conducted via the agriGO website. The 10 most significantly enriched GO terms in each of three main GO categories were presented (Additional file 1: Table S1). “oxidation-reduction process”, “hydrolase activity, hydrolyzing O-glycosyl compounds” and “catalase activity” were commonly found in the two *Elymus* species.

To characterize the complex biological behaviors of the transcriptome and further explore biological functions of differentially expressed transcripts, all the DETs were subjected to a KEGG pathway enrichment analysis (Additional file 2: Figure S1). I total, 1586 DETs could be annotated and assigned to KEGG pathway in *E. nutans*. The most representative pathways were “protein processing in endoplasmic reticulum” (64, 4.0%), “RNA transport” (51, 3.2%), “purine metabolism” (48, 3.0%), “peroxisome” (47, 2.9%) and “oxidative phosphorylation” (46, 2.9%). In *E. sibiricus*, 929 DETs were annotated and assigned to KEGG pathway. The most representative pathways were “carbon metabolism” (63, 6.8%), “biosynthesis of amino acids”(46, 4.9%), “protein processing in endoplasmic reticulum (31, 3.3%)” , “glycolysis/gluconeogensis” (30, 3.2%) and “oxidative phosphorylation” (29, 3.1%). Some DETs were annotated and assigned in other pathways such as “plant hormone signal transduction” and “pheylpropanoid biosynthesis”. For instance, 13 unigenes from abscission zone tissue in *E. sibiricus* were annotated in the pathway of “plant hormone signal transduction”. In the pathway of “pheylpropanoid biosynthesis”, 23 unigenes from abscission zone tissue in *E. nutans* were annotated and encoded 6 putative enzymes related to lignin biosynthesis.

### Comparative transcriptome analysis revealed candidate transcripts involved in seed shattering in two *Elymus* species

**Fig. 3:**
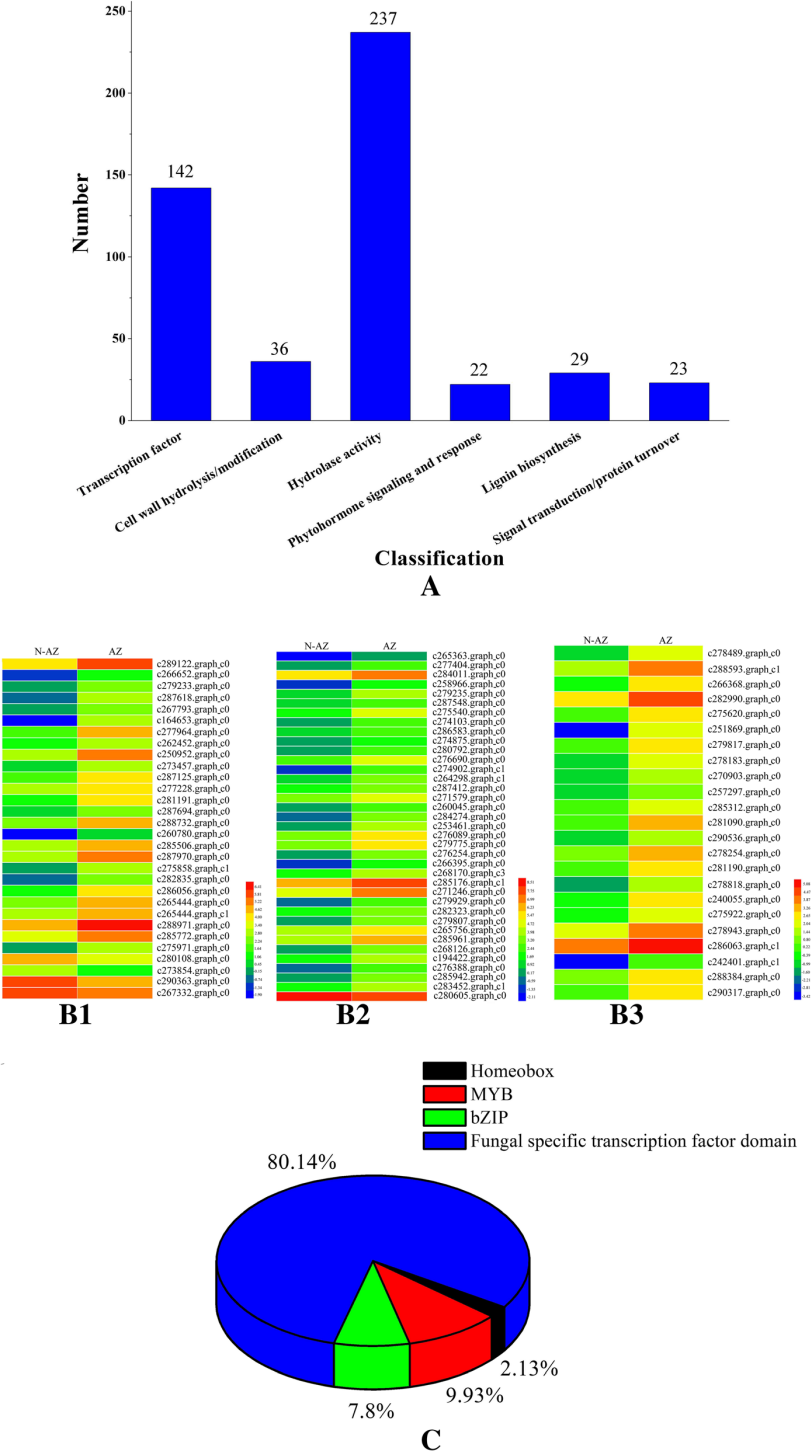
Candidate genes identified in abscission zone of *E. nutans.* **a** Six major function groups of the candidate genes based on annotation; **b** Heatmap diagram of the expression levels of candidate genes involved in lignin biosynthesis (B1), cell wall hydrolysis or modification (B2) and phytohormone signaling and response (B3). **c** The percentage of five types of transcription factor

To identify candidate genes involved in seed shattering in *E. nutans*, we further compared and analyzed 489 DETs between non-abscission zone (NAZ) and abscission zone (AZ) (Additional file 3: Table S2). Based on annotation, these DETs were divided into six major function groups: transcription factor (142), cell wall hydrolysis or modification (36), hydrolase activity (237), phytohormone signaling and response (22), lignin biosynthesis (29), signal transduction or protein turnover (23)(Fig. 3). The 142 transcription factor genes included 3 homeobox genes, 14 MYB genes, 11 bZIP genes and 114 fungal specific transcription factor, of which 139 genes were up-regulated in the abscission zone (Fig. 3c). Totally, 26 DETs involved in lignin biosynthesis were up-regulated in the abscission zone (Fig. 3, B1). A total of 36 DETs involved in cell wall hydrolysis or modification, of which 19, 9 and 7 genes involved in cellulase activity, polygalacturonase activity and pectin lyase activity were up-regulated in the abscission zone, respectively (Fig. 3, B2). In particular, all the DETs involved in phytohormone signaling and response were up-regulated in AZ compared to NAZ, of which 2, 4, 6, 4 and 7 involved in auxin, abscisic acid, ethylene, gibberellin, and cytokinine, respectively (Fig. 3, B3). To identify candidate genes related to abscission layers development, we further analyzed 116 DETs between 21 DAH and 28 DAH in *E. sibiricus* (Additional file 4: Table S3)*.* Based on annotation, these DETs were divided into five major function groups: transcription factor (47), cell wall hydrolysis or modification (23), phytohormone signaling and response (30), lignin biosynthesis (1), signal transduction (6), and peroxidase (9). The 47 transcription factor genes included 13 homeobox genes, 12 MYB genes, 16 bZIP genes, 3 MADS-box genes and 3 bHLH genes, no fungal specific transcription factor was found in *E. sibiricus* transcriptome data. 18 of 47 transcription factor genes were up-regulated in the abscission zone at 28 days after heading.

**Fig. 4:**
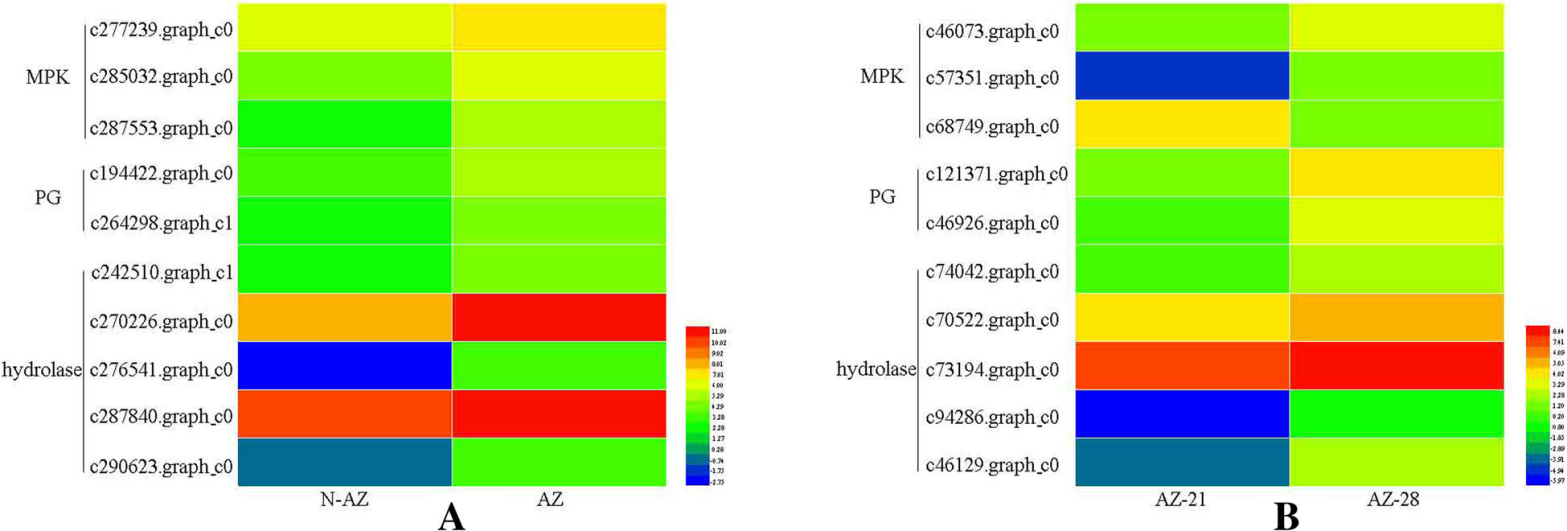
The different expression of 11 similar candidate genes in *E. nutans* AZ and NAZ (**a**), and *E. sibiricus* AZ at different development stage (**b**). These genes were involved in mitogen-activated protein kinase (MPK), polygalacturonase activity (PG) and hydrolase activity

We further compared and analyzed these DETs obtained in *E. nutans* with candidate genes in *E. sibiricus* transcriptome data. A total of 11 DETs were found similar with the DETs predicted from AZ-21 vs AZ-28 in *E. sibiricus* after sequence alignment, including 3 genes (c279235.graph_c0, c264298.graph_c1, c194422.graph_c0) involved in polygalacturonase activity, 5 genes (c287840.graph_c0, c242510.graph_c1, c270226.graph_c0, c290623.graph_c0, c276541.graph_c0) involved in hydrolase activity, and 3 genes (c285032.graph_c0, c277239.graph_c0, c287553.graph_c0) involved in mitogen-activated protein kinase. The 11 DETs were up-regulated in the abscission zone in *E. nutans* and differentially expressed in different developmental stages of abscission zone in *E. sibiricus,* suggesting these genes may be associated with abscission zone development and seed shattering (Fig. 4).

### Development of candidate gene-based EST-SSR markers and application in genetic diversity analysis

**Fig. 5:**
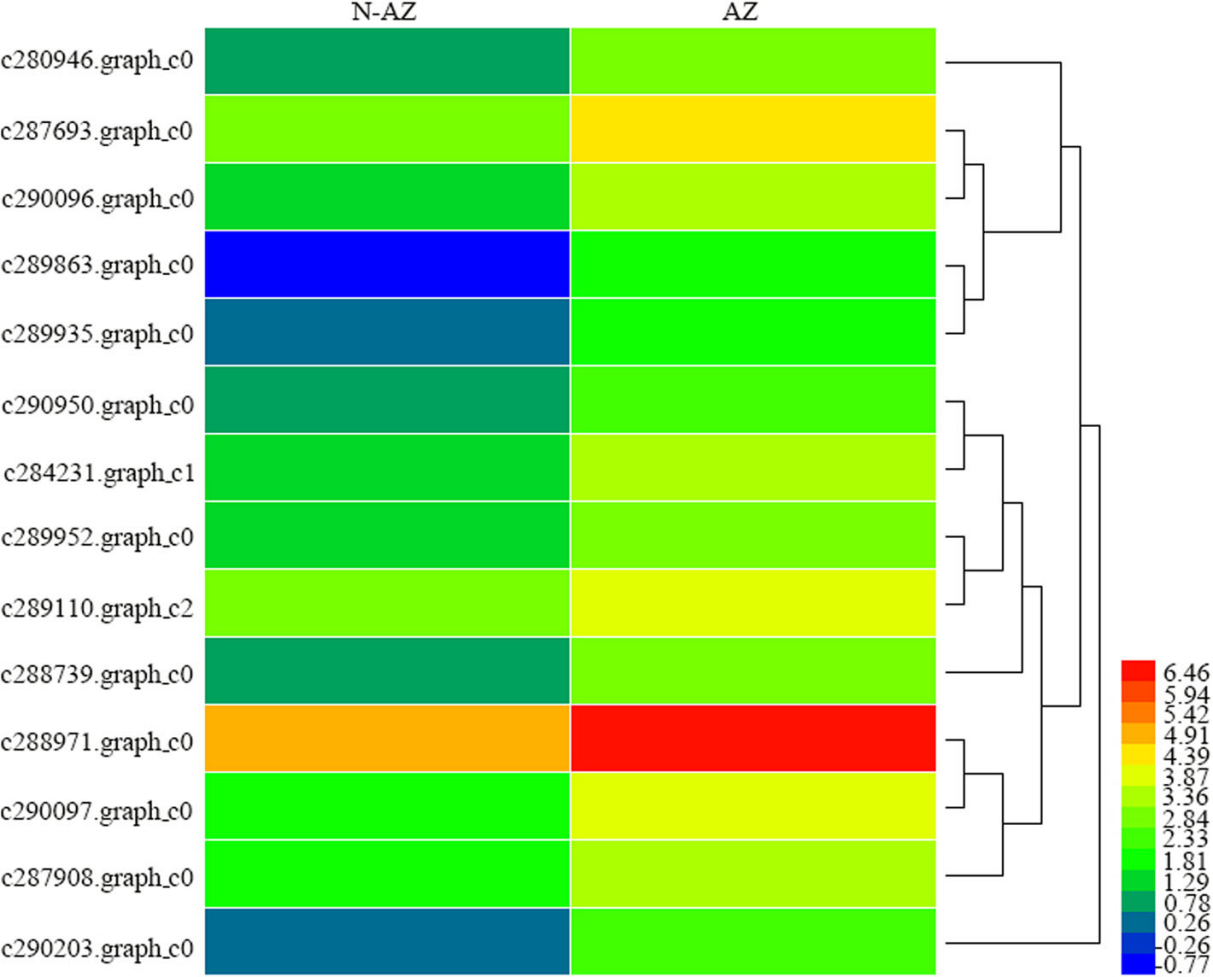
The different expressions of 14 candidate genes used for EST-SSR marker development

A total of 300 EST-SSR primer pairs were designed for candidate genes related to seed shattering (Additional file 5: Table S4). For validation, these primers were amplified in randomly selected five *E. nutans* genotypes, 67 primers of which were polymorphic. Finally, 14 polymorphic EST-SSR primers were used to study genetic diversity in 48 *E. nutans* genotypes. The 14 candidate genes used for EST-SSR markers development were up regulated in abscission zone (Fig. 5). Function annotation of these genes showed they were involved in hydrolase activity, transcription factor and lignin biosynthesis. In this study, a total of 175 bands were detected. The amplified bands per primer ranged from 7 (c287908.graph_c0, c284231.graph_c1, and c280946.graph_c0) to 18 (c290097.graph_c0 and c290950.graph_c0), with an average of 12.5. Polymorphic information content (PIC) varied from 0.220 (c287908.graph_c0) to 0.370 (c289935.graph_c0), with an average of 0.318 for this species (Table 6).

**Table 6:**
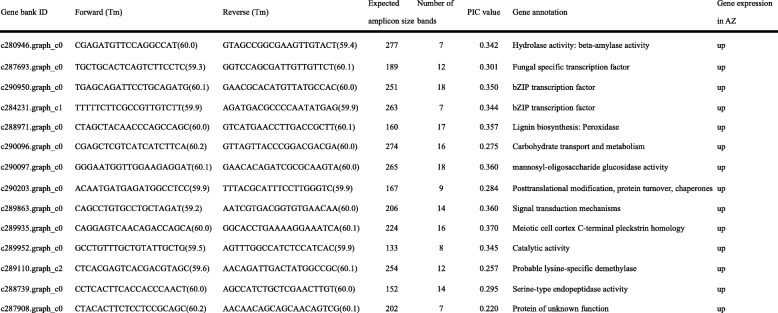
Candidate gene-based primer with Tm and molecular weight of expected band, amplified bands, PIC value and gene annotation

**Fig. 6:**
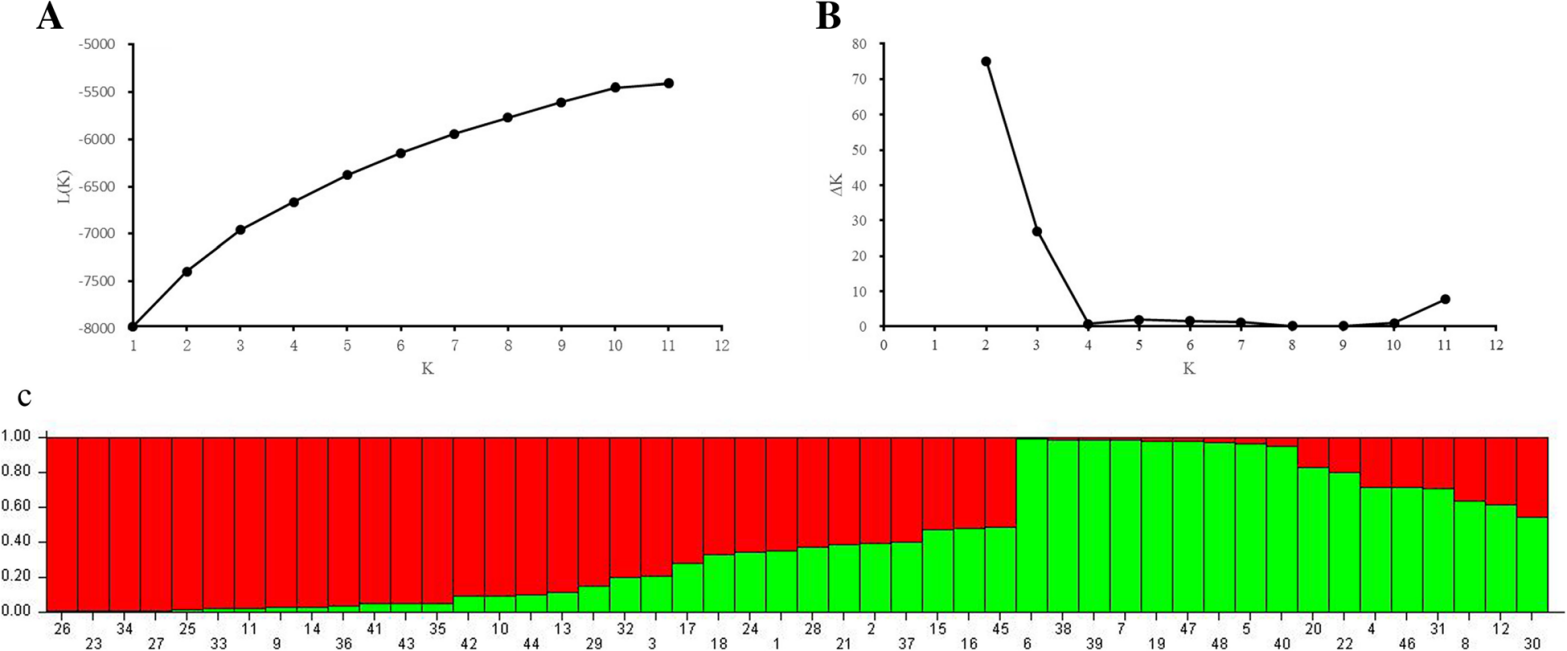
Population structure of 48 *E. nutans* genotypes inferred from STRUCTURE program with 14 gene-based EST-SSR markers data set. **a** Mean L (K) over 20 runs for each K value; **b** Maximum delta K (△K) values were used to determine the uppermost level of structure for K ranging from 2 to 11, here K is two and two clusters; **c** Two major groups of 48 *E. nutans* genotypes. The vertical coordinate of each group indicates the membership coefficients for each genotype. Different code and corresponding vertical lines represent individual genotype and different colors represent genetic stock

**Fig. 7:**
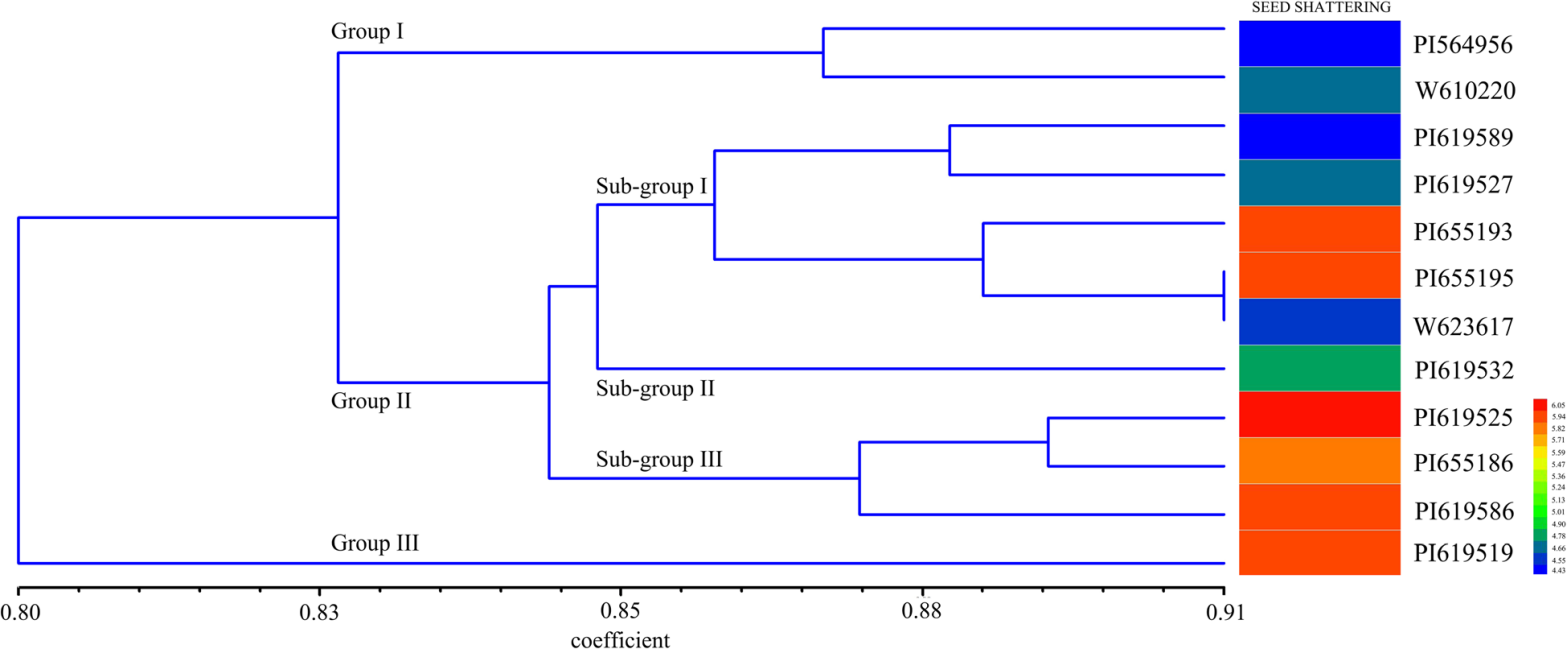
UPGMA-derived dendrogram of 12 accessions based on Jaccard’s genetic similarity and the Heatmap analysis of breaking tensile strength (BTS) value of the 12 accessions sequence, Tm, molecular weight of expected band

The STRUCTURE software was used to analyze the genetic structure of 48 genotypes. Based on the maximum likelihood and delta K (△K) values, the optimal number of groups was two for these genotypes (Fig. 6). Group 1 consisted of 31 genotypes and group 2 consisted of 17 genotypes. The UPGMA cluster analysis indicated that most accessions could be assigned into two major groups when genetic similarity value was 0.83 (Fig. 7). Group 1 contained two low seed shattering accessions (PI564956 and W610220). Group 2 were divided into two subgroups, three high seed shattering accessions (PI619525, PI 655186 and PI619586) were grouped together, the rest 6 accessions were assigned to the same subgroup. Interestingly, group 3 contained the highest seed shattering accession PI 619519. In general, accessions with similar seed shattering habit tended to group together. The developed EST-SSR markers from seed shattering candidate genes may have the potential to distinguish the genotypes with varied seed shattering habit.

## Discussion

### Cell wall degrading genes up-regulated in the abscission zone

Previous studies in major crops [16] and forage grasses like perennial ryegrass [6] have shown that seed abscission was generally caused by the development of abscission layers that were located in the rachilla just below each floret. Abscission is related to cleavage and degradation of cell wall component. A correlation between seed shattering and the degree of degradation for abscission layers was found, suggesting increased hydrolytic enzymes activity in abscission zone contribute to high seed shattering [10, 11]. Our transcriptome analysis revealed that 36 DETs for cell wall hydrolysis or modification were up-regulated in the abscission zone. These include genes for cellulase (CE), polygalacturonase (PG) and pectate lyase. CE and PG have a crucial effect on the degradation of abscission layer, which then cause the shattering of seed and other organs [34, 35]. Accompanying organ separation is an increase in the activity of several cell wall hydrolytic enzymes including CE and PG. High seed shattering accessions had higher CE and PG activity in abscission zone at seed maturity stage [10, 11]. Cellulase is the first hydrolytic enzyme reported, which plays a critical role in plant cell wall loosening during plant organ abscission [36]. In rice, *OsCel9D* gene, encoding an endo-1,4,-*β*- glucanase gene, plays an important role in modifying cell wall structure and component during abscission, and mutations of this gene hamper the abscission process in seed shattering through reducing cell elongation and cellulose content, and increasing the pectin content [37]. Polygalacturonase is another important enzyme that hydrolyzes cell wall pectin. PG has been demonstrated to promote cell separation, abscission in various plant organs like leaf, flower, fruit and seed [38, 39]. The PG genes were strongly up-regulated at the onset of abscission in tomato pedicels [40] and citrus leaves [41]. In addition, the high expression level of PG genes in the abscission zone was related to high seed shattering in *E. sibiricus* [11]. Several previous reports also indicated the potential roles of pectate lyases in abscission process as pectate lyases could depolymerize pectins by catalyzing the eliminative cleavage of a-1,4-linked galacturonic acid. For floral organ, petal abscission requires extensive dissolution of the middle lamella which is rich in pectins, activation of the pectate lyase is considered to be an important step towards dissolution of the middle lamella [42]. Many Arabidopsis pectate lyase-like genes are required for numerous aspects of growth and development [43].

### A complex regulation of plant hormone pathway during abscission

Plant hormones like abscisic acid, ethylene and cytokinine play an important role in regulating a wide range of plant growth and development processes [10]. Ethylene is known to be important regulator of abscission of plant organ such as flowering, leaf and seeds [44]. The high level of ethylene in plant organ is commonly associated with tissue senescence and cell stress [45]. The ethylene insensitive mutant of *Arabidopsis etr1* exhibited a delay in the shedding of floral parts [46]. In the present study, we found that 5 ethylene receptor genes (*ETR1*) were up-regulated in abscission zone when compared with non-abscission zone, suggesting the roles of these ethylene response genes in regulating abscission. In addition, we also found that 7 genes involved in cytokinine were up-regulated in abscission zone. Cytokinine, a plant growth regulator has an influence on the activity of some enzymes involved in metabolism. The activity of esterases, a hydrolytic enzyme significantly increase under the influence of cytokinine [47]. The expression of the number of pectinesterases increases during organ shedding [48]. Furthermore, a abscission-related transcriptome analysis in the tomato flower abscission zone revealed that a wide variety of genes for phytohormone signing were up-regulated during abscission [40]. Shedding of plant organ including leaves, buds, petal, fruit and seeds is a complex and highly coordinated process involving multiple changes in abscission zone development, plant hormone level, metabolism and gene expression [11, 49]. Based on our current data, it is difficult to identify the key hormone genes that regulate and determine abscission process. A balance and interaction of these plant hormones may be the key factor.

### Transcription factors genes

Transcription factors are essential players in the signal transduction pathways, and orchestrate gene expression control of a cell [50]. In the present study we found 142 transcription factor genes. Many reported seed shattering genes in rice are transcription factors genes like *qSH1* and *SH4. qSH1* is BEL1-type homeobox gene [14], and *SH4* encodes a transcription factor with a Myb 3 DNA binding domain [13]*.* Many MYB proteins act as critical components of multiple hormone-mediated transcriptional cascades, including ethylene, auxin, abscission acid, which regulate plant organ abscission [51]. In the study, 3 homeobox genes and 14 MYB genes were up-regulated in the abscission zone. Also, bZIP transcription factors are important members of transcription factor families*.* Several bZIP transcription factors genes like *OsABF2* [52] and *OsbZIP23* [53] were reported to be responsible for hormone signal. Previously*,* several TGA-type bZIP genes were suggested to regulate the expression of genes involved in abscission [54]. The up-regulation of 11 bZIP genes in abscission zone of *E. nutans* may act as positive regulator. Most reported fungal-specific transcription factors influence plant pathogenicity. The fungal-specific transcription factor-encoding gene *Vdpf* was shown to be associated with vegetative growth and virulence in *Verticillium dahliae* [55]. Fungal-specific transcription factor *AbPf2* activates pathogenicity in *Alternaria brassicicola* [56]. The previous study also reported that the fungal-specific transcription factors are not restricted to strictly fungal-specific functions. This means that some of the general functions of other transcription factors have been transferred at some moment of evolution to fungal-specific transcription factors [50]. Interestingly, 114 of 142 transcription factor genes are fungal specific transcription factors that specifically expressed in abscission zone, indicating these genes might have effect on abscission zone development and degradation. However, the potential role of fungal-specific transcription factors in seed abscission in *E. nutans* remains largely unknown*.*

### Candidate genes-based EST-SSR marker for marker assisted selection for seed shattering

The development of novel *Elymus* germplasm with improved seed shattering depends on the accurate evaluation of seed shattering. The accuracy of morphological identification is usually affected by environmental factors. Compared with morphological identification, DNA markers closely linked to important agronomic traits have enormous potential to improve the precision of trait selection and the breeding efficiency via marker-assisted selection (MAS) [57]. To date, diverse molecular markers like amplified fragment length polymorphisms (AFLP), RFLP (restriction fragment length polymorphism), inter simple sequence repeat (ISSR), sequence related amplified polymorphism (SRAP), start codon targeted (SCoT) and single nucleotide polymorphism (SNP) have been developed. Among all, simple sequence repeat (SSR) markers are abundant, co-dominant, high reproducibility, and highly polymorphic. Traditional SSR markers developed from random genomic sequence have uncertainty of linkage with the functional genes, whereas candidate gene sequence based SSR or EST-SSRs have better possibility of linkage to agronomically important loci [58]. Therefore, development of SSR markers based on candidate genes related to particular trait may greatly facilitate marker assisted selection in breeding programme for desired trait. Xiao et al. [59] developed 182 gene-based SSR markers related to cold tolerance in oil palm by exploiting transcriptome data, and suggested these SSR markers would be particularly useful for gene mapping and population structure analysis in oil palm germplasm with different cold response. Tranbarger et al. [60]. developed SSR markers based on putative genes associated with post-transcriptional and transcriptional regulatory functions during growth development of *Elaeis guineensis*. These polymorphic markers provided tools for molecular breeding strategies. Molla et al. [61] identified and analyzed 19 novel salt responsible candidate genes based SSRs from rice. Dendrogram based on molecular data showed these markers could distinguish salt susceptible and salt tolerant genotypes. In this study, 14 seed shattering candidate genes involved in bZIP transcription factor, hydrolase activity, and lignin biosynthesis were up regulated in abscission zone, indicating they may be specifically or strongly related with AZ development or seed shattering. Previous studies showed bZIP transcription factors during abscission may regulate downstream processes mostly related to ABA [50]. A previous transcriptome analysis in *E. sibiricus* reported many genes involved in lignin biosynthesis were differentially expressed in abscission zone [9]. In rice, the BEL1-type homeobox gene *SH5* could inhibit lignin biosynthesis, overexpression of this gene in the non-shattering variety led to an increase in seed shattering because of lower lignin level in the basal region of spikelets [16]. The 14 candidate genes based EST-SSR markers were evaluated and validated for genetic diversity in 6 high seed shattering and 6 low seed shattering *E. nutans* accessions. The dendrogram revealed most accessions with similar seed shattering degree tended to group together. The results indicated these markers had the potential to be used as the novel and remarkable candidate for diversity analysis among *E. nutans* accessions with different seed shattering habits.

## Conclusions

In this study we described RNA-sequencing for abscission zone and non-abscission zone differentiation in *E. nutans* and AZ development of 21 and 28 days after heading in *E. sibiricus.* In addition, polymorphic candidate gene-based EST-SSR markers were developed and characterized. Sequencing results showed that 7644 DETs were predicted between AZ and NAZ tissues of *E. nutans,* among which 489 candidate genes were identified. Especially, eleven similar candidate genes involved in polygalacturonase activity, hydrolase activity, and mitogen-activated protein kinase were up-regulated in the abscission zone of the two *Elymus* species. These transcripts provide hypotheses for further testing and development of low shattering *Elymus* germplasm. At the same time, 14 polymorphic candidate gene-based EST-SSR markers were finally used to study genetic diversity of *E. nutans* accessions with different seed shattering degree. These results showed EST-SSR loci related to candidate genes may have potential application in identifying trait-associated markers in *E. nutans.*

## Additional files

Additional file 1: **Table S1.** Significantly enriched Go terms found in differentially expressed transcript sets of two *Elymus* species. (XLS 33 kb)

Additional file 2: **Figure S1.** KEGG classification results of differentially expressed transcripts (DETs) found in *E. nutans* (A) and *E. sibiricus* (B)*. (PDF 214 kb)*

Additional file 3: **Table S2.** Differentially expressed transcripts related to seed shattering found in abscission zone and non-abscission zone. (XLS 357 kb)

Additional file 4: **Table S3.** Differentially expressed transcripts related to seed shattering found in abscission zone at 21 days (AZ-21) and 28 days (AZ-28) after heading. (XLS 165 kb)

Additional file 5: **Table S4.** Gene-based EST-SSR primer information: reverse and forward. (XLS 188 kb)

## Abbreviations

AZ: Abscission zone
BTS: Breaking tensile strength
CE: Cellulase
COG: Cluster of orthologous group
DAH: Days after heading
DET: Differentially expressed transcript
FDR: False discovery rate
FPKM: Fragments per kilobase per million fragments mapped
GO: Gene ontology
KEGG: Kyoto encyclopedia of genes and genomes
KOG: Eukaryotic orthologous groups
Nr: Non-redundant protein sequence
Pfam: Protein family
PG: Polygalacturonase
qRT-PCR: Quantitative real-time PCR
QTL: Quantitative trait locus
SNP: Single nucleotide polymorphism
Swiss-Prot: Annotated protein sequence database
