# Supplementary material for: Elymus nutans genes for seed shattering and candidate gene-derived EST-SSR markers for germplasm evaluation
Source: BMC Plant Biol. 2019 Mar 13;19:102. doi: 10.1186/s12870-019-1691-4 (PMC6416926; doi:10.1186/s12870-019-1691-4)
Supplement: Supplementary file 2 — Figure S1. KEGG classification results of differentially expressed transcripts (DETs) found in E. nutans (A) and E. sibiricus (B). (PDF 214 kb) [file 12870_2019_1691_MOESM2_ESM.pdf]

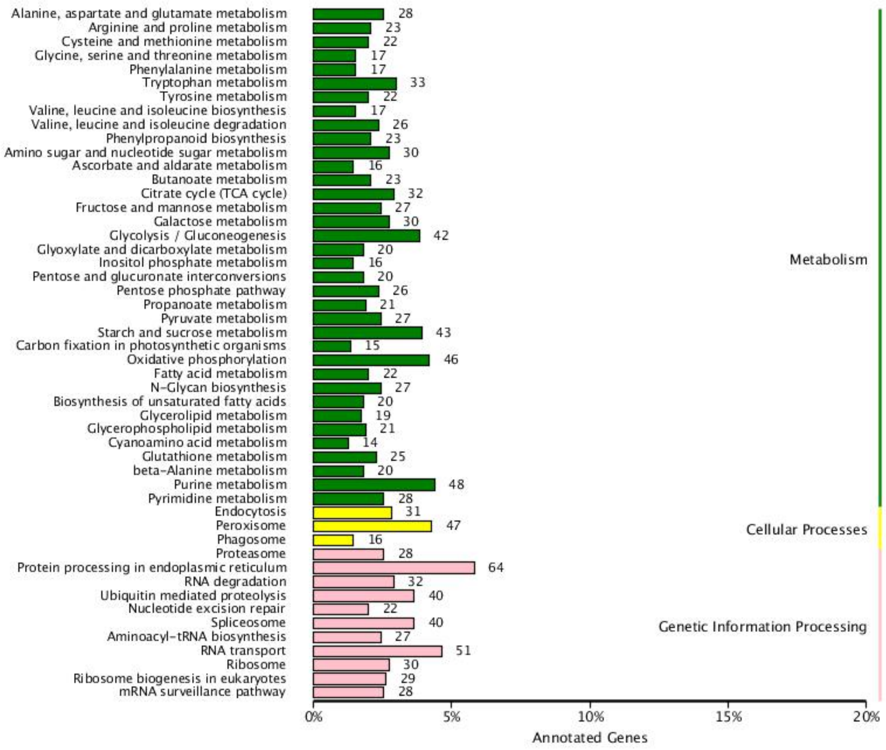

A. KEGG enrichment analysis results of DEGs in *E. nutans.* All DETs were assigned to three categories: metabolism, cellular processes and genetic information processing

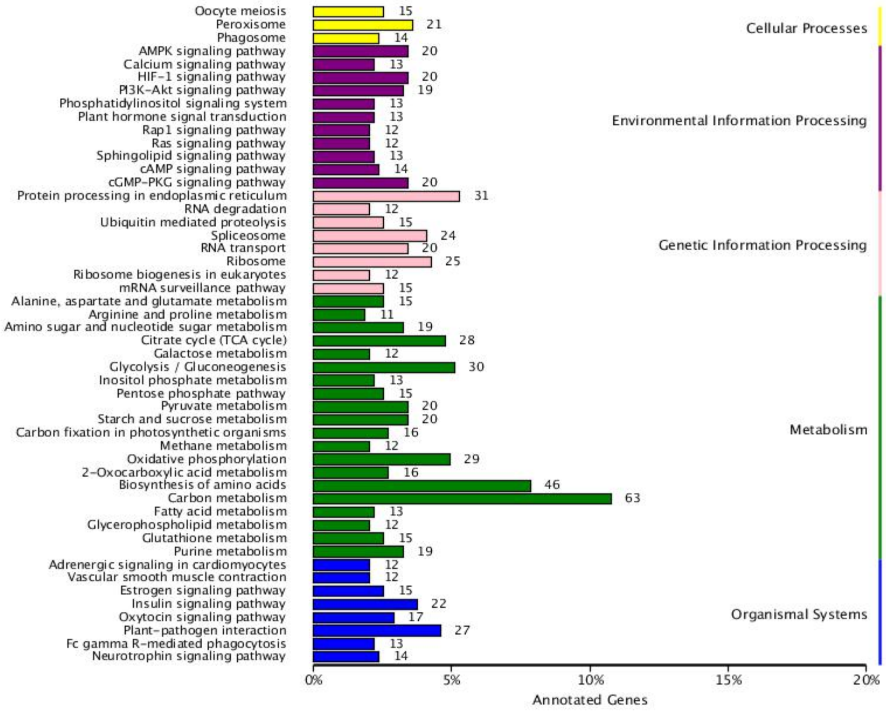

B. KEGG enrichment analysis results of DETs in *E. sibiricus.* All DETs were assigned to five categories: cellular process, environmental information processing, genetic information processing, metabolism and organismal systems.
